# Application value of multi-gene mutation detection in the clinical management of pediatric papillary thyroid carcinoma: a preliminary exploration

**DOI:** 10.3389/fendo.2024.1405142

**Published:** 2024-06-05

**Authors:** Yuguo Wang, Hui Wang, Gongxun Tan, Xinping Wu, Bin Wang, Zhihan Tan, Jing Du, Xiuying Li, Ying Xu, Na Yan, Xiaoqin Qian

**Affiliations:** ^1^Department of Ultrasound, Traditional Chinese Medicine Hospital of Nanjing Lishui District, Nanjing, Jiangsu, China; ^2^Affiliated Hospital of Integrated Traditional Chinese and Western Medicine, Nanjing University of Chinese Medicine, Nanjing, Jiangsu, China; ^3^Jiangsu Province Academy of Traditional Chinese Medicine, Nanjing, Jiangsu, China; ^4^Department of Endocrinology, Yancheng City No.6 People’s Hospital, Yancheng, Jiangsu, China; ^5^Department of Ultrasound, Affiliated People’s Hospital of Jiangsu University, Zhenjiang, Jiangsu, China; ^6^Department of Ultrasound, Jiangsu Hospital of Integrated Traditional Chinese and Western Medicine, Nanjing, Jiangsu, China; ^7^Nanjing Dian Diagnostics Group Co., Ltd., Nanjing, Jiangsu, China; ^8^Key Laboratory of Digital Technology in Medical Diagnostics of Zhejiang Province, Dian Diagnostics Group Co., Ltd., Hangzhou, Zhejiang, China

**Keywords:** PTC, pediatric, molecular markers, BRAF, RET/PTC, molecular assisted diagnosis

## Abstract

**Objectives:**

Thyroid cancer rarely occurs in children and adolescents. Molecular markers such as *BRAF*, *RAS*, and *RET/PTC* have been widely used in adult PTC. It is currently unclear whether these molecular markers have equivalent potential for application in pediatric patients. This study aims to explore the potential utility of a multi-gene conjoint analysis based on next-generation targeted sequencing for pediatric papillary thyroid carcinoma (PTC).

**Materials and methods:**

The patients diagnosed with PTC (aged 18 years or younger) in the pediatrics department of Lishui District Hospital of Traditional Chinese Medicine were retrospectively screened. A targeted enrichment and sequencing analysis of 116 genes associated with thyroid cancer was performed on paraffin-embedded tumor tissues and paired paracancerous tissue of fifteen children (average age 14.60) and nine adults (average age 49.33) PTC patients. Demographic information, clinical indicators, ultrasonic imaging information and pathological data were collected. The Kendall correlation test was used to establish a correlation between molecular variations and clinical characteristics in pediatric patients.

**Results:**

A sample of 15 pediatric PTCs revealed a detection rate of 73.33% (11/15) for driver gene mutations *BRAF V600E* and *RET* fusion. Compared to adult PTCs, the genetic mutation landscape of pediatric PTCs was more complex. Six mutant genes overlap between the two groups, and an additional seventeen unique mutant genes were identified only in pediatric PTCs. There was only one unique mutant gene in adult PTCs. The tumor diameter of pediatric PTCs tended to be less than 4cm (p<0.001), and the number of lymph node metastases was more than five (p<0.001). Mutations in specific genes unique to pediatric PTCs may contribute to the onset and progression of the disease by adversely affecting hormone synthesis, secretion, and action mechanisms, as well as the functioning of thyroid hormone signaling pathways. But, additional experiments are required to validate this hypothesis.

**Conclusion:**

*BRAF V600E* mutation and *RET* fusion are involved in the occurrence and development of adolescent PTC. For pediatric thyroid nodules that cannot be determined as benign or malignant by fine needle aspiration biopsy, multiple gene combination testing can provide a reference for personalized diagnosis and treatment by clinical physicians.

## Introduction

Thyroid cancer is a relatively rare malignant tumor in the population under 18 years old (1). The SEER Cancer Statistics Reviews (https://seer.cancer.gov/archive/csr/1975_2016/index.html) show an increasing trend in thyroid cancer incidence among children between 1975 and 2016. From 1975 to 1995, the annual growth rate of thyroid cancer in the pediatric population was a modest 0.8%. However, from 1996 to 2016, this growth rate increased significantly to an annual rate of 4.6%. The predominant pathological subtype is papillary thyroid carcinoma (PTC) (2).

Assessment and management recommendations for thyroid cancer in children are derived from adult thyroid cancer guidelines. Given the rapid evolution of molecular biology, there has been a substantial increase in the application of molecular diagnostic technology in precision medicine, especially in molecular-assisted diagnosis and therapeutic strategies for tumor management. Molecular diagnostic techniques also serve as vital tools in the comprehensive management of thyroid cancer (3). The National Comprehensive Cancer Network (NCCN) guidelines (4) and the 2015 American Thyroid Association (ATA) guidelines (5) for adult thyroid cancer recommend the use of molecular markers such as *BRAF*, *RAS*, and *RET/PTC* to supplement the diagnosis of benign and malignant thyroid nodules that cannot be accurately identified through fine needle aspiration biopsy (FNAB). However, the clinical application value of these molecular markers has not been clearly defined in the diagnosis and treatment guidelines for pediatric thyroid cancer (6).

In this study, we attempted to evaluate the potential clinical utility of multi-gene conjoint analysis in a limited number of pediatric PTCs. We wanted to enhance the understanding of gene variation characteristics and clinical management of pediatric thyroid cancer.

## Methods

### Patients and tumor samples

**Figure 1:**
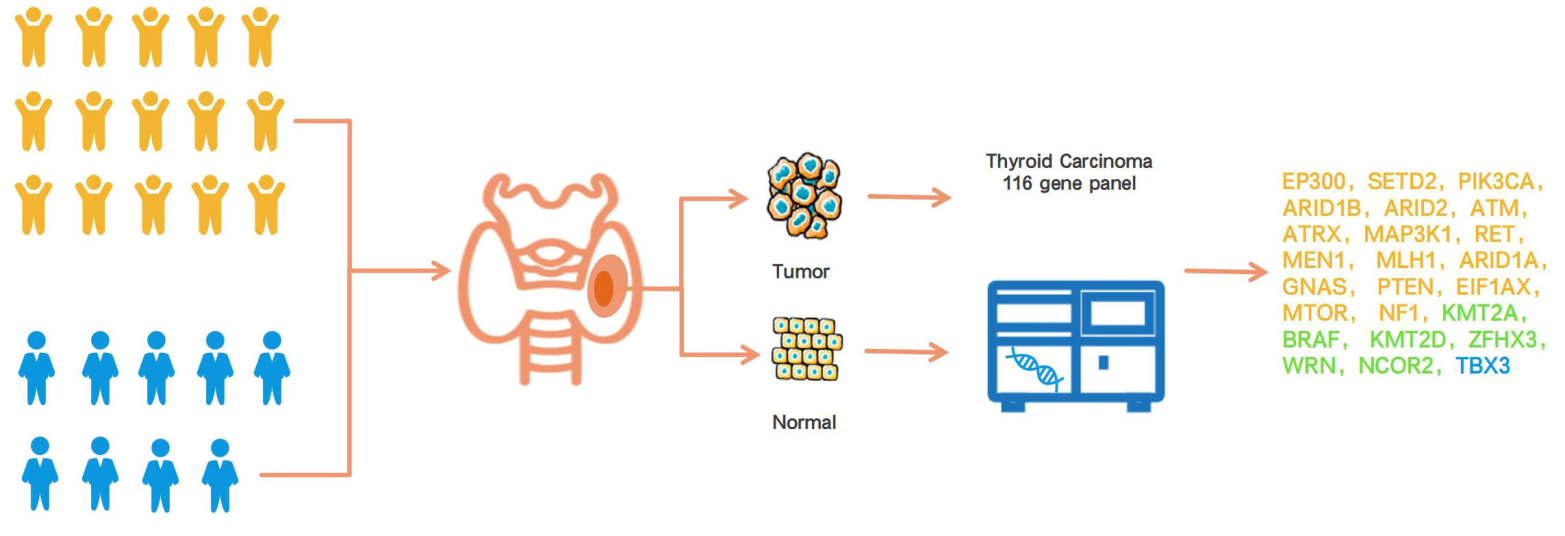
Overview of this research design. The yellow icon represents the children’s group, and the blue icon means the adult group. The gene color detected only in the child group was yellow, the gene color detected only in the adult group was blue, and the gene color detected in both the child and adult groups was green.

We searched for pediatric patients (age ≤ 18 years old) in the Traditional Chinese Medicine Hospital of Lishui District and screened the patients participating in the study according to the following inclusion and exclusion criteria. Inclusion criteria: 1) Imaging suggested the presence of thyroid nodules. 2) Surgical resection was performed at the Traditional Chinese Medicine Hospital of Lishui District for treatment from September 2019 to September 2023. 3) Postoperative pathological diagnosis was papillary thyroid carcinoma. 4) The complete medical records consist of general patient information such as age, gender, etc., and imaging records that highlight tumor size and location, extrathyroidal extension (ETE), multi-focal nature of the tumor, blood flow signals, capsule integrity, and cancer morphological characteristics, etc. 5) Preserve sufficient amounts of paraffin-embedded papillary thyroid carcinoma tissue specimens and adjacent tissue specimens after surgical resection. The preservation period should not exceed three years. 6) All test samples adequately satisfied the quality control requirements of gene sequencing experiments. Exclusion criteria: 1) Postoperative pathological diagnosis was unclear. 2) Previous history of thyroid surgery or radiation therapy. 3) family history of thyroid surgery and thyroid tumors. 4) Patients whose tissue samples have failed repeatedly. 5) A history of malignancy or severe disease in other systems. A cohort of 15 children who met the pre-determined inclusion and exclusion criteria were selected from the pool of 24 children who underwent surgical intervention within a recent three-year period. We collected postoperative paraffin-embedded cancer tissue specimens and paracancerous tissue from each pediatric PTC patient. The proportion of tumor cells in cancer tissue samples is at least 20%. The paraffin-embedded tissue section should meet the requirements of a tissue embedding area of 1X1cm^2^, with a thickness greater than 3um and five slices. Cancer tissue and paracancerous tissue were used for the sequencing analysis of 116 thyroid cancer-related genes. Cancer tissue serves as the target detection specimen, while paracancerous tissue serves as paired controls in order to obtain pure somatic mutation information in cancer tissue. At the same time, we recruited nine surgically treated PTC patients to participate in the sequencing analysis of 116 thyroid cancer genes (**Figure 1**). The selection criteria and tissue specimen requirements for adult and children PTC patients are consistent. This study has been approved by the Ethics Committee of Lishui District Traditional Chinese Medicine Hospital (No.2022LW037).

### Thyroid cancer 116 gene panel

The sequencing analysis in this study covered 116 genes closely related to thyroid cancer, including all exons of 63 genes and DNA base sequences of the TERT gene promoter region, used to detect single nucleotide variations (SNV) and short segment insertion or deletion variations (InDel); At the same time, RNA base sequences covering 56 genes were used to detect known and unknown gene fusion breakpoints (Three genes coexist between DNA and RNA sequencing genes). Genes were selected from diagnostic and treatment guidelines in China and other countries, the 5th edition of the World Health Organization (WHO) classification of thyroid tumors, expert consensus, public databases (OncoKB, TCGA, Cosmic, Clinvar, My Cancer Genome, Genomenon, etc.), and cutting-edge literature (**Supplementary Table 1**).

### Targeted sequencing analysis

The proportion of tumor cells in all tumor tissue specimens is not less than 20%. Nucleic acid extraction from cancerous and para-cancerous tissues was performed as described in Qiagen’s AllPrep FFPE DNA/RNA Kit. A sequencing library was constructed using the hybridization capture method with a starting amount of 20–40ng genomic DNA and cDNA for targeted enrichment of 116 genes related to thyroid cancer. All tests were conducted in a laboratory that extensively evaluated and approved by the College of American Pathologists (CAP). The library sequencing was accomplished with an Illumina Nextseq 500 instrument, designed and manufactured by Illumina, Inc., in San Diego, California, USA. The mean sequencing depth was calculated to be a minimum of 500X, with a minimum frequency of variation of 1%. The sequencing data was first converted to FASTQ format, and then the human genome was meticulously mapped to the human reference sequence (hg19) through the Burrows-Wheeler aligner. Subsequently, genomic variant annotation was performed using the state-of-the-art ANNOVAR 21 software.

### Mutation characteristic analysis and statistical analysis

All analyses were conducted using the R programming language, version 4.1.1, released on August 10, 2021. GenVisR visualized the landscape of gene variation. The cluster profiler package was used to perform functional enrichment analysis of Gene Ontology (GO) and pathway enrichment analysis of Kyoto Encyclopedia of Gene and Genomes (KEGG). Kendall’s ratio was used for correlation analyses. Continuous variables were reported using mean and standard deviation values. Subgroup analyses were conducted through Fisher’s exact test. All tests were evaluated with a 2-sided significance level of P ≤ 0.05.

## Result

### Clinicopathological characteristics of pediatric PTC patient

In the pediatric PTC patients, female patients were 1.5 times more likely than male patients. The median age of PTC in children is 15 years, with the youngest patient being 7 years old and the oldest 18 years old. The median age of adult PTC is 47, with a minimum age of 34 and a maximum age of 63. Unilateral involvement was typically observed in about 80% of cases. Almost half observed multiple lesions. In most cases, pediatric PTC tumors were identified as measuring less than 4 cm in diameter (86.67%). About half of pediatric PTC patients have irregular tumor morphology, blurred edges, and abundant blood flow signals. One-third of cases reported the presence of ETE. Lymph node metastasis occurred in four out of five patients. The above characteristics of children were not independently present in the data of this study compared to adult PTC patients. We found that the tumor size in all adult PTC patients was greater than 4cm, but in the case of pediatric PTC, it was the opposite. The tumor size in 86.67% of pediatric PTC patients was less than 4cm (**Table 1**, **Supplementary Table 2**).

**Table 1:** Comparison between clinical and pathological characteristics in pediatric and adult PTC patients.

Clinical characteristics	Pediatric PTC	Adult PTC	*P-value*
**Gender**			1
Male	6/15 (40.00%)	3/9 (33.33%)	
Female	9/15 (60.00%)	6/9 (66.67%)	
**Tumor sides differences**			0.74
Left	5/15 (33.33%)	3/9 (33.33%)	
Right	7/15 (46.67%)	3/9 (33.33%)	
Bilateral	3/15 (20.00%)	3/9 (33.33%)	
**Multifocality**			1
Single lesion	8/15 (53.33%)	4/9 (44.44%)	
Multiple lesions	7/15 (46.67%)	5/9 (55.56%)	
**Tumor size (cm)**			4.21E-05
≤4	13/15 (86.67%)	0	
>4	2/15 (13.33%)	9/9 (100.00%)	
**Morphology rules**			0.38
Yes	8/15 (53.33%)	2/9 (22.22%)	
No	7/15 (46.67%)	6/9 (66.67%)	
unknown	0	1/9 (11.11%)	
**Edge blur**			0.43
Yes	7/15 (46.67%)	6/9 (66.67%)	
No	8/15 (53.33%)	3/9 (33.33%)	
**Internal echo**			0.72
Low	7/15 (46.67%)	6/9 (66.67%)	
Medium	2/15 (13.33%)	0	
High	3/15 (20.00%)	3/9 (33.33%)	
Mix	1/15 (6.67%)	0	
unknown	2/15 (13.33%)	0	
**Homogeneous echo**			1
Yes	1/15 (6.67%)	0	
No	13/15 (86.67%)	8/9 (88.89%)	
unknown	1/15 (6.67%)	1/9 (11.11%)	
**Increased blood flow within the nodule**			0.05
Yes	7/15 (46.67%)	6/9 (66.67%)	
No	7/15 (46.67%)	0	
unknown	1/15 (6.67%)	3/9 (33.33%)	
**LN metastasis**			
Yes	12/15 (80.00%)	2/9 (77.78%)	1
No	3/15 (20.00%)	2/9 (22.22%)	
< 5	1/12 (8.33%)	7/7 (100.00%)	1.59E-04
≥ 5	11/12 (91.67%)	0	
**Distant metastasis**			1
Yes	1/15 (6.67%)	0	
No	12/15 (80.00%)	9/9 (100.00%)	
unknown	2/15 (13.33%)	0	
**Extra thyroidal extension, ETE**			0.35
Yes	5/15 (33.33%)	1/9 (11.11%)	
No	10/15 (66.67%)	8/9 (88.89%)	

### Molecular characteristics of pediatric PTC

**Figure 2:**
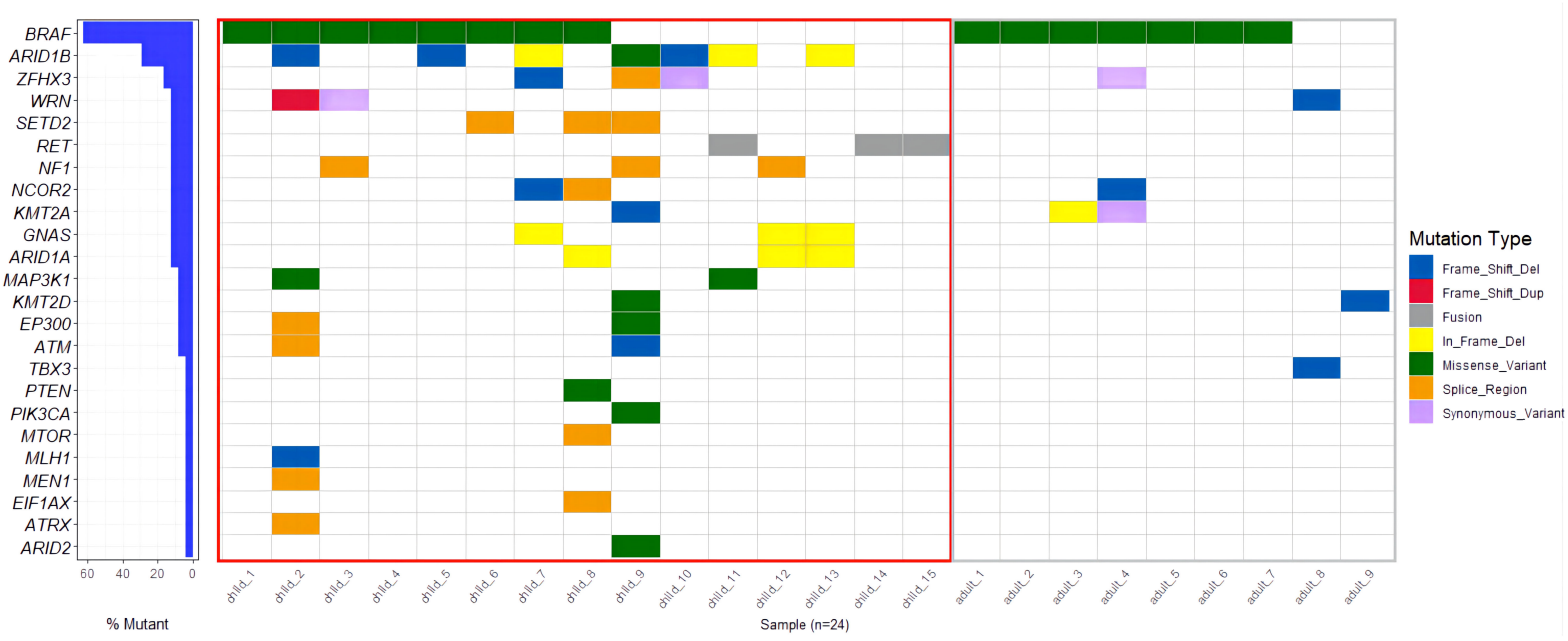
Overview of gene mutation profiles of pediatric PTCs and adult PTCs.

**Figure 3:**
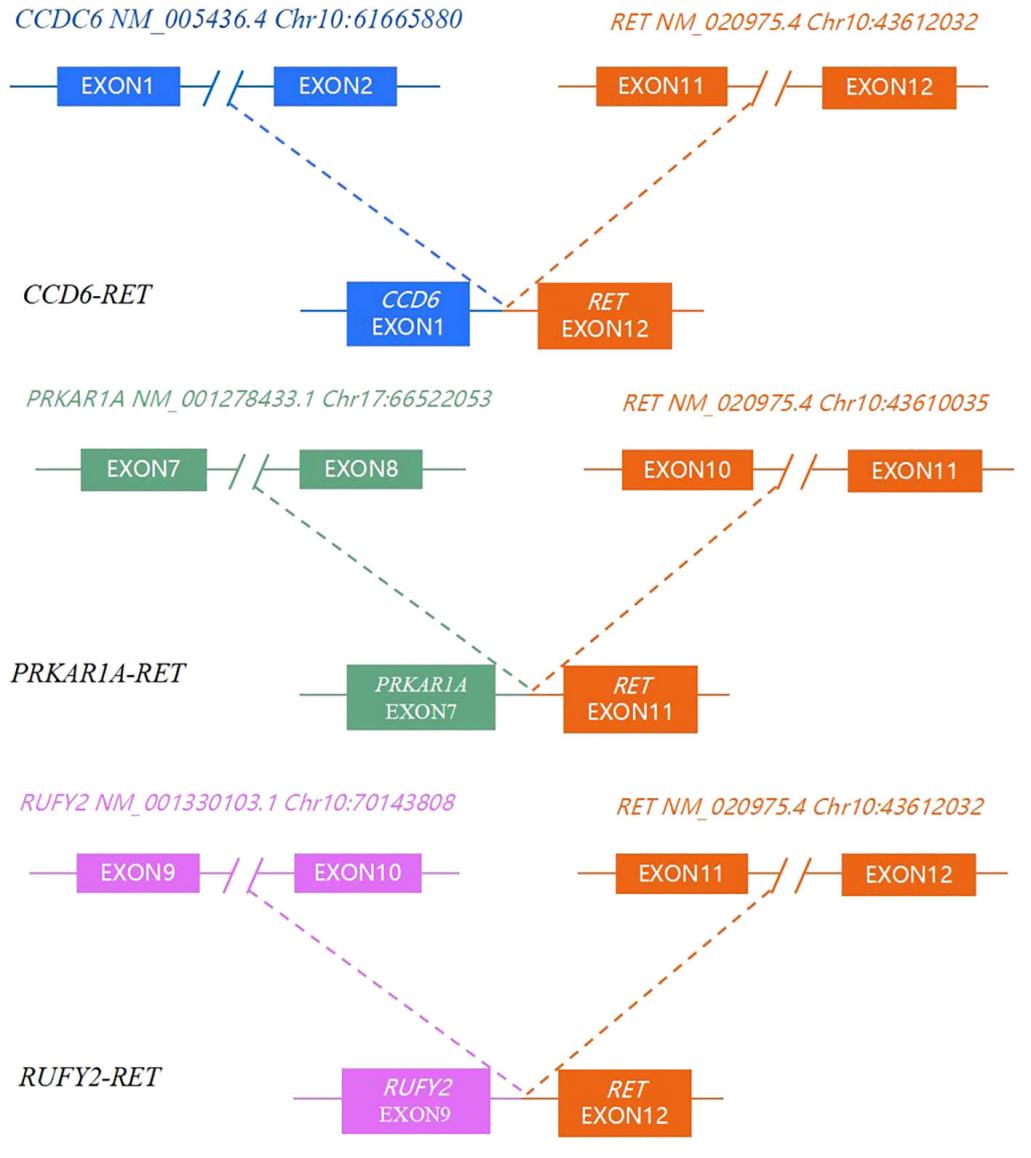
RET fusion type, partner gene and breakpoint location.

A total of 23 genes with molecular variations were detected in pediatric PTC patients (**Figure 2**, **Supplementary Table 3**). The *BRAF* gene exhibited the highest mutation rate, with an observed frequency of 53.33%(8/15). Notably, all identified mutations in the *BRAF* gene were the *BRAF V600E* subtype. The mutation rate of *ARID1B* (7/15, 46.67%) was second only to *BRAF V600E*. *RET* fusion (3/15, 20%) was detected in 3 patients. The *RET* fusion variation and *BRAF V600E* mutation were mutually exclusive (**Figure 2**). The fusion types of *RET* were *CCD6 (Chr10:61665880):: RET (Chr10:43612032)*, *PRKAR1A (Chr17:66522053):: RET (Chr10:43610035)*, and *RUFY2 (Chr10:70143808):: RET (Chr10:43612032)* (**Figure 3**). *PRKAR1A* and *RUFY2* were rare fusion partner genes. The *RAS* gene was not found to harbor any mutations in 15 cases of pediatric PTC. However, *RET* fusion mutation and *BRAF V600E* mutation accounted for 73.33% of pediatric PTC patients (11/15). This suggested that if FNA cannot determine thyroid nodule malignancy in children, thyroid cancer driver gene testing has the potential to assist in diagnosis.

**Figure 4:**
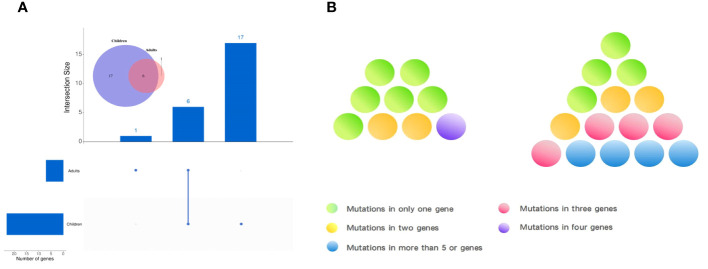
Characteristics of gene mutations in pediatric PTCs and adult PTCs. **(A)** Common or unique mutated genes between pediatric PTCs and adult PTCs. **(B)** Genetic mutation complexity diagram of pediatric PTCs (right) and adult PTCs (left).

Observing the genetic mutations of pediatric PTCs and adult PTCs, it was found that pediatric PTC patients have unique genetic mutation characteristics. The genetic mutation profile of pediatric PTC patients was rich and complex (**Figure 2**). From the comprehensive analysis of gene mutations, 23 distinct genes were altered in pediatric PTCs. Adult PTCs only detected mutations in 7 genes. The overlap analysis of gene mutations between pediatric PTCs and adult PTCs (**Figure 4A**) showed that there were a total of 6 mutated genes in the two groups. The pediatric PTC group had up to 17 unique mutated genes, while the adult PTC group had only one unique mutated gene. The pediatric PTC group almost covers the mutant genes of the adult PTC group, with unique mutant genes accounting for 70.83% of all mutant genes. Based on analysis of composite gene mutation levels carried by individual patients (**Figure 4B**), 66.67% of adult PTC group detected only one mutated gene. The mutation profile of the adult PTC group was convergence and simplicity, with 77.78% of patients experiencing *BRAF V600E* mutations. However, the proportion of compound gene mutations in the pediatric PTC group was as high as 73.33%.

### Functional enrichment analysis of mutant genes detected only in pediatric PTC patients

In order to further understand the unique mutation gene functions of pediatric PTC populations, we conducted a functional enrichment analysis. In the analysis of KEGG signaling pathway enrichment (**Supplementary Figure 1**), we observed that mutant genes unique to pediatric PTC patients were significantly enriched in pathways such as hormone synthesis, secretion, and action, thyroid hormone signaling, platinum resistance, endocrine resistance, tumor PD-L1 expression, and PD-1 checkpoint, etc. The altered genes identified in pediatric PTC may result in abnormalities in the production, release, and regulation of growth hormone and thyroid hormone, thereby promoting the development of PTC and potentially influencing the efficacy of specific alternative therapeutic strategies. Gene Ontology (GO) analysis showed that the unique mutant genes of pediatric PTC patients were significantly enriched in epithelial cell development, DNA metabolism regulation, protein binding specific sites, immune system processes, and responses to DNA damage stimuli, etc (**Supplementary Figure 2**).

### Correlation analysis of mutant genes and clinical indicators in pediatric PTC

**Figure 5:**
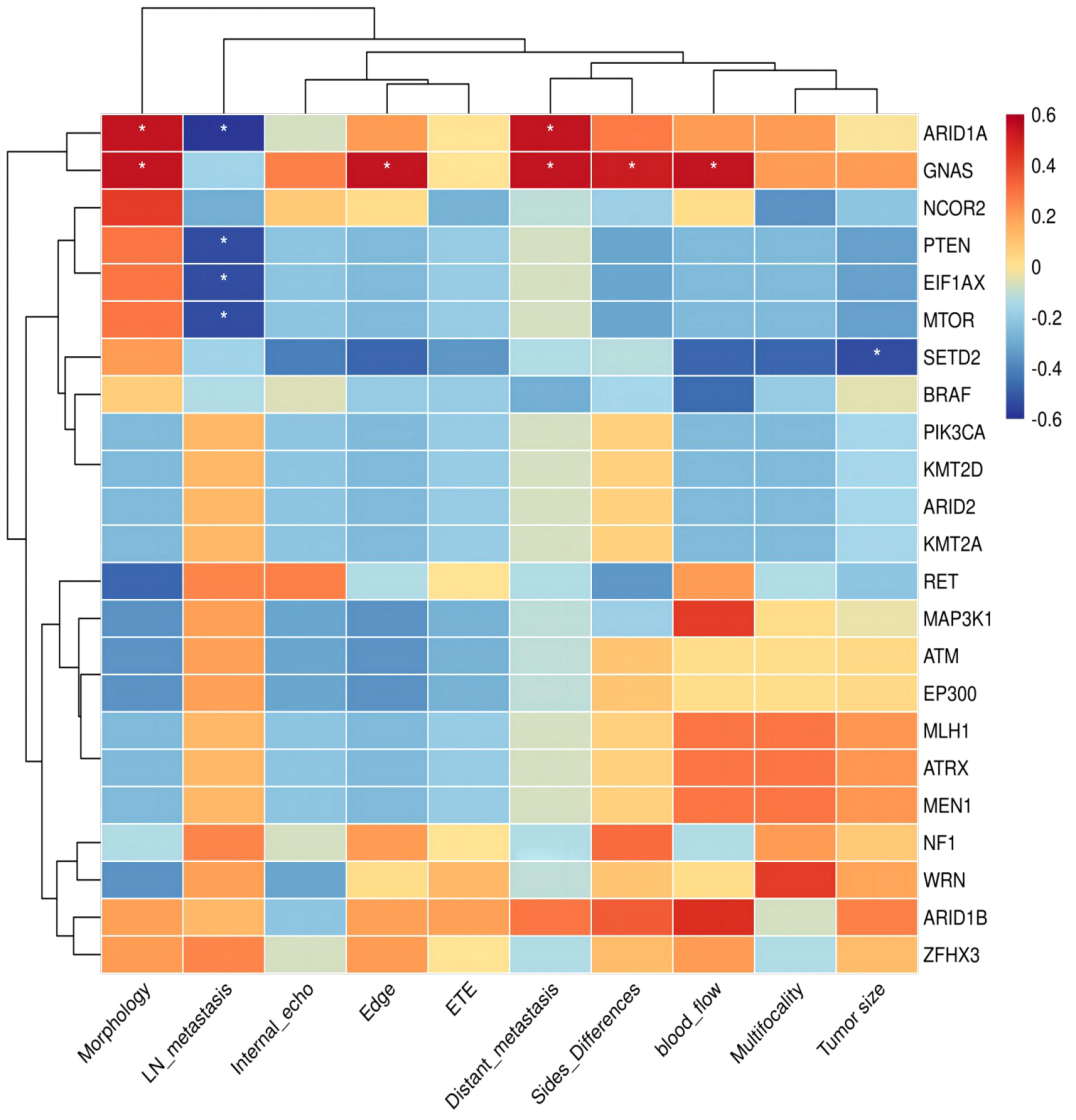
Kendall correlation between specific genes of pediatric PTCs and clinical indicators. The Kendall correlation coefficient was determined by a two-tailed significance test at a significance level of 0.05. The results of this test are presented in the figure using an asterisk (*) to indicate p-values below 0.05.

The biology of *GNAS* mutation showed an elevated level of invasiveness, characterized by irregular tumor shape, ambiguous tumor boundaries, multiple nodules affecting both lobes or the entire thyroid gland, and distant metastases (p<0.05, **Figure 5**). Patients with the *ARID1A* mutation often have irregular tumor shapes (p<0.05). In a case of pediatric PTC with negative lymph nodes, mutations were simultaneously identified in *PTEN*, *EIF1AX*, and *MTOR*. However, we did not observe a positive or negative correlation between RET fusion and clinical indicators in the Kendall correlation test. Significantly, it was observed that the lymph nodes from *RET* fusion patients were found to be positive, with an elevated number of metastatic nodes exceeding eight. One individual even exhibited 35 metastatic lymph nodes.

## Discussion

In pediatric patients, thyroid nodules typically present as painless masses that are incidentally identified during routine physical examinations (6). It is estimated that 1–3% of all pediatric patients suffer from thyroid nodules, and a staggering 22–26% of these pediatric patients may suffer from malignant thyroid nodules (7–9). Therefore, it is important to distinguish between benign and malignant thyroid nodules in clinical practice. FNAB is widely regarded as the decisive method for preoperative pathological diagnosis of thyroid nodules, which can distinguish the nature of benign and malignant nodules (10). The Bethesda System for Reporting Thyroid Cytopathology (TBSRTC) provides a fundamental foundation for the diagnosis and categorization of FNAB cellular pathology. It categorizes thyroid fine needle aspiration (FNA) into six different classes, each associated with varying levels of risk for the presence of malignant tumors (10, 11). Bethesda III and Bethesda IV cannot reliably classify thyroid cytology results as benign or malignant (10, 11). However, Bethesda III and Bethesda IV collectively account for 40% of all FNA diagnoses in children (ranging from 13% to 43%) (12). Some studies have shown that thyroid nodules in Bethesda III and Bethesda IV are also associated with an increased risk of malignancy in children (ranging from 28% to 58%) (12).

Molecular-assisted diagnosis has been recognized in the management of adult thyroid diseases. The role of molecular detection in thyroid lesions in children is constantly being studied and explored. We reviewed twenty-two studies of molecular variation in PTC patients aged eighteen years and younger (**Table 2**). These studies explore the molecular variations of any one or combination of *BRAF V600E*, *RET* fusion, and *RAS* mutations in pediatric PTC patients. A total of eighteen studies identified mutations in the thyroid cancer driver gene *BRAF V600E*, with a collective sample seven hundred and seventy-one patients. Of these, two hundred and thirty-four patients with *BRAF V600E* mutations demonstrated a mutation positivity rate of 30.35% (234/771) (**Supplementary Figure 3A**). Twelve studies investigated the mutation-positive rate of *RET* fusion in pediatric PTC. Out of three hundred and nineteen PTC patients under eighteen years old, *RET* fusion was identified in eighty-five patients, translating to a fusion-positive rate of 26.65% (85/319). A total of four hundred and twenty-two pediatric PTC patients were examined for *RAS* mutations in twelve studies. Of these, twenty-two patients (5.21%) were identified to have positive *RAS* mutations. In our preliminary study of 15 children with PTC (**Supplementary Figure 3B**), a substantial portion of these patients, 53.33% (8/15), were found to carry *BRAF V600E* mutation. This is higher than the overall *BRAFV600E* mutation rate of 30.35% (234/771) in previous studies we reviewed. But we also found that the mutation rates of *BRAF V600E* in small sample studies by Ballester et al. (22) and Prasad et al. (24) were 10/23 (43.48%) and 13/27 (48.15%), respectively (**Table 2**). A study of *BRAF V600E* mutations in Chinese pediatric PTC patients showed a mutation rate of 57.39% (97/169) (31). This may be a characteristic of PTC molecular mutations in Chinese children, but the limitations of the small sample size should also be considered. There may be bias in the results, and it is necessary to expand the sample size to verify this preliminary exploration result. In the literature review, the incidence of *RET* fusion mutation was 26.65% (85/319). During our test, we observed that the mutation rate for *RET* fusion was 20% (3/15). It is noteworthy that 2 out of 3 *RET* fusions have rare fusion partners, namely *PRKAR1A* and *RUFY2*. *PRKAR1A (Chr17:66522053):: RET (Chr10:43610035), and RUFY2 (Chr10:70143808):: RET (Chr10:43612032)* both retained the tyrosine kinase domain (exon 12–19). For patients with *RET/PTC* rearrangement who are ineffective in both surgical treatment and radioactive iodine therapy and still have significant disease progression, the possibility of targeted therapy may be considered. The *RAS* gene mutation in the literature summary only appeared in 5.21% (22/422) of pediatric PTC patients. Our testing data did not detect patients with *RAS* gene mutations. In our retrospective study, thirteen studies detected *RAS* gene mutations, of which six studies were all negative for *RAS* gene mutations. (**Table 2**) According to adult PTC studies, hot spot mutations in the driving genes of thyroid cancer are mutually exclusive. This study analyzed a total of fifteen pediatric patients, among which eleven had identified *BRAF V600E* or *RET/PTC* rearrangements. The remaining four patients did not present any *RAS* mutations, possibly due to the small number of patients. The combined incidence of molecular variation among the three driving genes in nine studies simultaneously covering *BRAF V600E*, *RET* rearrangement, and *RAS* mutations was 50.19%, more than half (**Supplementary Figure 3C**). Among our 15 pediatric PTC patients, 73.33% patients had driver gene molecular variations. This may suggest that molecular testing could provide reference information for more than half of children with thyroid cancer. In addition, we distinguished pediatric PTC patients based on whether driver genes (*BRAF V600E/RAS/RET*) were mutated. We found that Pediatric PTCs in the *BRAF V600E/RAS/RET* negative group (simultaneous negative) tend to have a single lesion with irregular morphology but clear tumor margins. Pediatric PTCs in the *BRAF V600E/RAS/RET* positive group (one of them was positive) likely exhibit multiple lesions with regular morphology and blurred boundaries (**Supplementary Table 4**).

**Table 2:** Review of molecular variations related to pediatric thyroid cancer.

Researches	Age	PTC No.	*RET* Fusion	*RAS* Mutation	*BRAF V600E*
Williams et al. (13)	7–14 years	21	10/21 (47.62%)	——	——
Lima et al. (14)	<18 years	17	——	——	1/17 (5.88%)
Elisei et al. (15)	<18 years	25	10/25 (40%)	——	——
Motomura et al. (16)	9–14 years	10	3/10 (30%)	——	——
Rosenbaum et al. (17)	10–17 years	20	——	——	4/20 (20.00%)
Sassolas et al. (18)	≤18 years	28	8/28 (28.57%)	1/28 (3.57%)	2/28 (7.14%)
Pauws et al. (19)	9–16 years	8	——	0	——
Givens et al. (20)	<18 years	19	——	——	7/19 (36.84%)
Onder et al. (21)	≤18 years	50	——	——	15/50 (30.00%)
Ballester et al. (22)	10–18 years	23	5/23 (21.74%)	0	10/23 (43.48%)
Picarsic et al. (23)	<18 years	17	3/17 (17.65%)	3/17 (17.65%)	3/17 (17.65%)
Prasad et al. (24)	6–18 years	27	6/27 (22.22%)	0	13/27 (48.15%)
Nikita et al. (25)	7–18 years	32	6/32 (18.75%)	1/32 (3.13%)	9/32 (28.13%)
Gertz et al. (26)	8–18 years	14	2/14 (14.29%)	0	5/13 (38.46%)
Cordioli et al. (27)	4–18 years	30	——	——	3/30 (10.00%)
Mostoufi-Moab et al. (28)	≤18 years	59	12/59 (20.34%)	3/59 (5.08%)	12/59 (20.34%)
Wasserman et al. (29)	<18 years	28	7/28 (25.00%)	0	5/28 (17.86%)
Sisdelli et al. (30)	≤18 years	77	——	12/77 (15.58%)	14/77 (18.18%)
Yangsen Li et al. (31)	≤18 years	169	——	——	97/169 (57.39%)
Alzahrani et al. (32)	9–18 years	52	——	——	12/52 (23.06%)
Cordioli et al. (33)	≤18 years	35	13/35 (37.14%)	0/35 (0%)	3/35 (8.57%)
Alzahrani et al. (34)	≤18 years	74	——	2/79 (2.53%)	19/79 (24.05%)

We need to acknowledge that there are some limiting factors in this small sample study of pediatric thyroid cancer. First, due to the low incidence of thyroid cancer in children, we only obtained 15 pediatric PTC patients who were available for the study. The small sample size does not allow age stratification of pediatric PTC patients, and molecular variation characteristics of pediatric PTC patients in different age groups cannot be observed. In order to better understand and validate the clinical characteristics, molecular variation patterns, and characteristics of pediatric PTC, it is necessary to expand the sample size, go beyond the small scope of the initial study, and conduct comprehensive exploration and detailed analysis in multiple hospitals to avoid potential result bias due to sample size reasons. Furthermore, this study is limited by the inherent limitations of the targeted sequencing methodology, which may not be sufficient to comprehensively identify all potential molecular variations in pediatric PTC patients. In addition, this study lacked follow-up data for pediatric PTC patients, which precluded the study from examining the relationship between molecular mutation traits and prognosis.

In conclusion, by using NGS sequencing technology and multi-gene joint detection, our study identified a positive rate of 73.33% for *BRAF V600E* and *RET* rearrangement of the PTC driver gene in 15 pediatric PTC patients. Multi-gene joint testing can further stratify uncertain FNAB diagnoses, suggesting the potential utility of molecular testing as an auxiliary diagnostic tool for pediatric thyroid nodules.

## Data availability statement

The data presented in the study are deposited in the CNSA repository (https://db.cngb.org/cnsa/), accession number CNP0005254.

## Ethics statement

The studies involving humans were approved by Lishui District Traditional Chinese Medicine Hospital (No.2022LW037). The studies were conducted in accordance with the local legislation and institutional requirements. Written informed consent for participation in this study was provided by the participants’ legal guardians/next of kin.

## Author contributions

YW: Writing – review & editing, Writing – original draft, Project administration, Investigation. HW: Writing – review & editing, Writing – original draft, Methodology. GT: Writing – review & editing, Writing – original draft, Data curation. XW: Writing – review & editing, Investigation. BW: Writing – review & editing, Data curation. ZT: Writing – review & editing, Data curation. JD: Writing – review & editing, Data curation. XL: Writing – review & editing, Methodology. YX: Writing – review & editing, Methodology. NY: Writing – review & editing, Visualization, Investigation, Conceptualization. XQ: Writing – review & editing, Conceptualization.

## Supplementary material

Supplementary Figure 1 Functional enrichment analysis of KEGG genes specific to pediatric PTCs.

Supplementary Figure 2 Functional enrichment analysis of GO genes specific to pediatric PTCs.

Supplementary Figure 3 Review of molecular alterations in pediatric thyroid carcinoma. **(A)** To summarize BRAF V600E, RET rearrangement, and RAS mutations rates in 22 studies of thyroid cancer in children. **(B)** The molecular variability rates of BRAF V600E, RET rearrangement, and RAS mutations in 15 pediatric patients in this study. **(C)** A total of 10 molecular variation studies covering BRAF V600E, RET rearrangement, and RAS mutations were conducted in 291 patients. It demonstrated BRAF V600E, RET rearrangement, and RAS mutation rates.
