# Supplementary figures and images for: Application value of multi-gene mutation detection in the clinical management of pediatric papillary thyroid carcinoma: a preliminary exploration
Source: Front Endocrinol (Lausanne). 2024 Jun 5;15:1405142. doi: 10.3389/fendo.2024.1405142 (PMC11188297; doi:10.3389/fendo.2024.1405142)

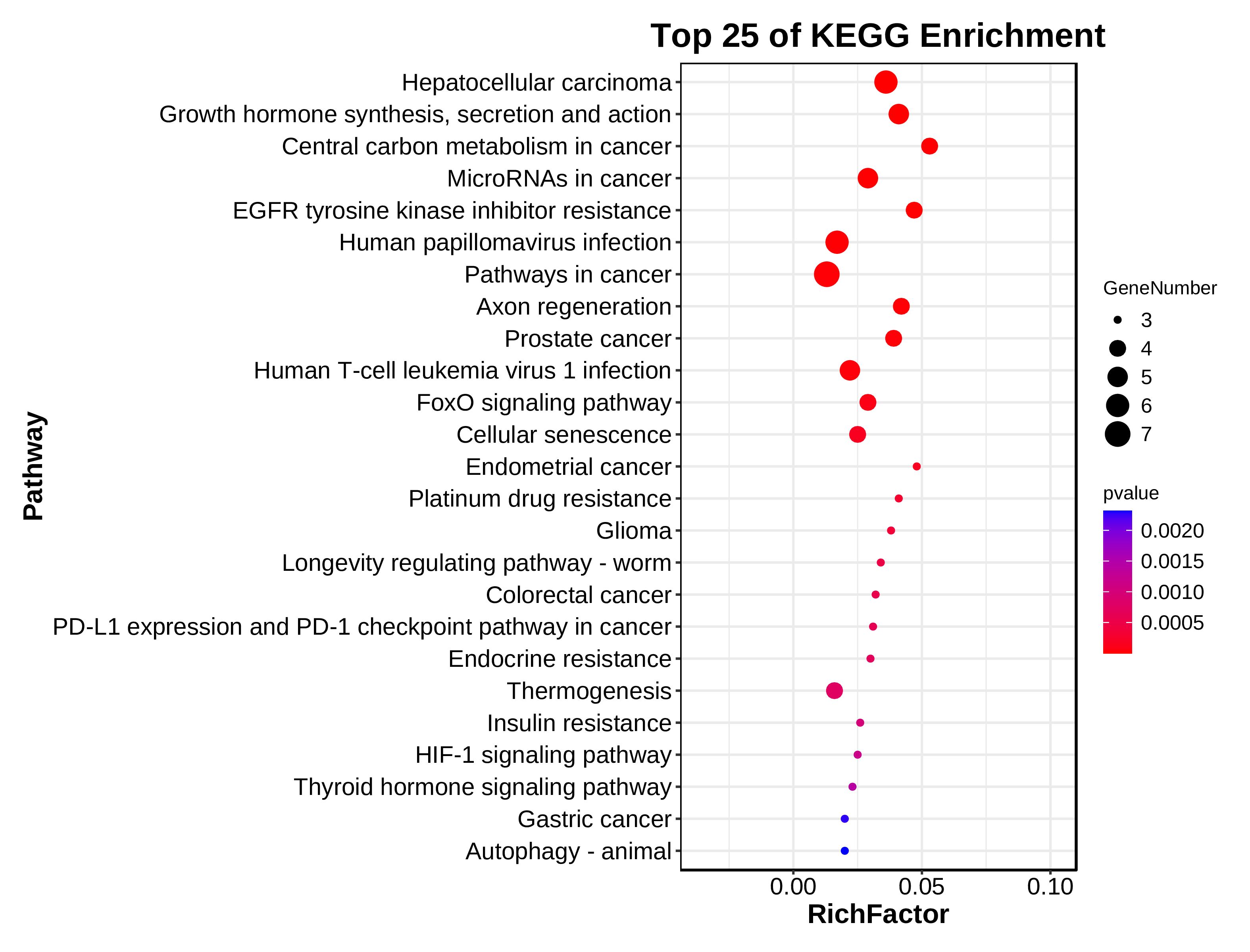

Supplement: Supplementary Figure 1 — Functional enrichment analysis of KEGG genes specific to pediatric PTCs. [file Image_1.png]

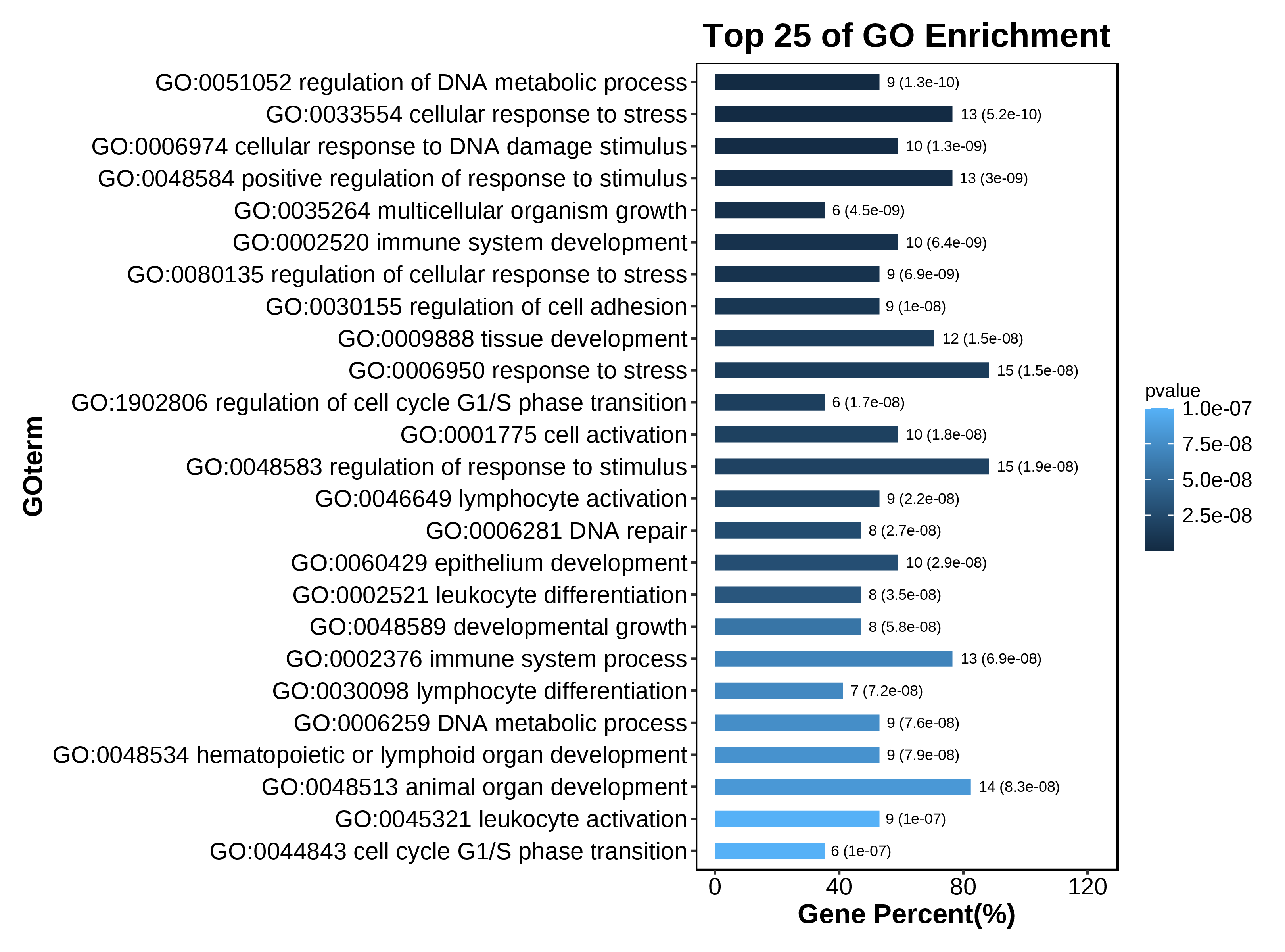

Supplement: Supplementary Figure 2 — Functional enrichment analysis of GO genes specific to pediatric PTCs. [file Image_2.png]

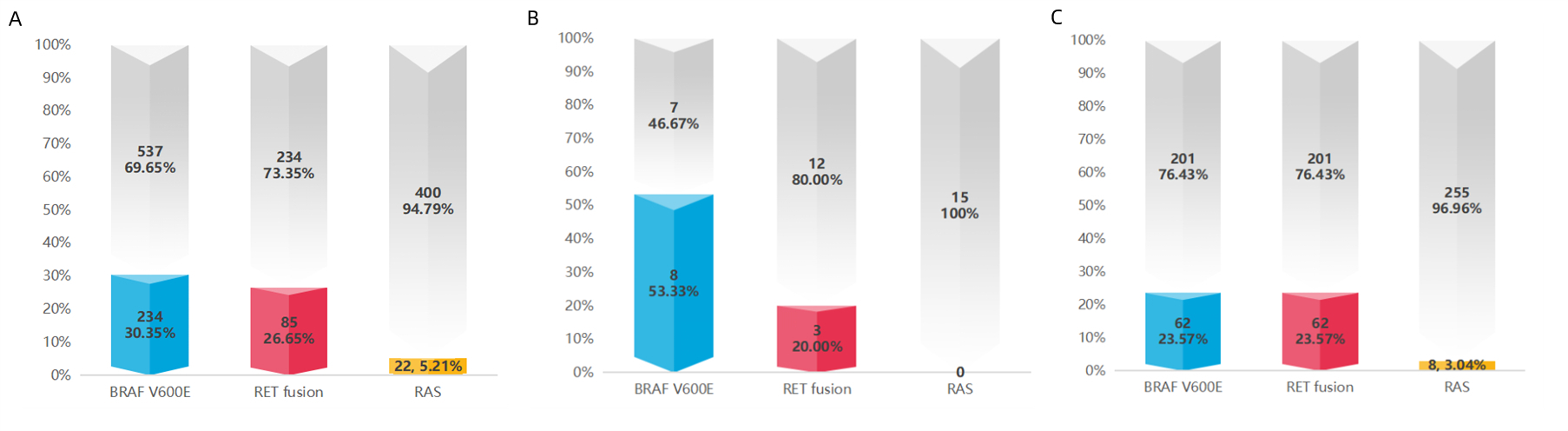

Supplement: Supplementary Figure 3 — Review of molecular alterations in pediatric thyroid carcinoma. (A) To summarize BRAF V600E, RET rearrangement, and RAS mutations rates in 22 studies of thyroid cancer in children. (B) The molecular variability rates of BRAF V600E, RET rearrangement, and RAS mutations in 15 pediatric patients in this study. (C) A total of 10 molecular variation studies covering BRAF V600E, RET rearrangement, and RAS mutations were conducted in 291 patients. It demonstrated BRAF V600E, RET rearrangement, and RAS mutation rates. [file Image_3.jpeg]
